# Resistant Epilepsy and Developmental Delay in a Syndromic Infant: A Case of Congenital Disorder of Glycosylation Type Ik From India

**DOI:** 10.7759/cureus.107892

**Published:** 2026-04-28

**Authors:** Shambhavi Trivedi, Jyoti C Suvarna, Durga Patel, Purnima Nair, Salil Vaniawala, Pramila Menon, Vasundhara Tamhankar, Parag M Tamhankar

**Affiliations:** 1 Pediatrics, Holy Spirit Hospital, Mumbai, IND; 2 Genetics, SN Gene Lab Pvt Ltd, Surat, IND; 3 Pediatrics, Dr. D. Y. Patil Medical College, Hospital, and Research Centre, Pune, IND; 4 Genetics, Centre for Medical Genetics, Mumbai, IND

**Keywords:** alg1, autosomal recessive disorders, congenital disorder of glycosylation, from india, whole-exome sequencing (wes)

## Abstract

Type I congenital disorder of glycosylation (CDG) is a group of genetic disorders that affect the biosynthesis of dolichol-linked oligosaccharides or the transfer of oligosaccharides onto nascent glycoproteins. Mutations in the *ALG1* gene cause CDG type Ik. We report a ten-month-old female with CDG type Ik presenting with recurrent seizures, global developmental delay, hypotonia, and dysmorphic features. Whole-exome sequencing revealed a homozygous likely pathogenic variant chr16:5071980G>T or c.131G>T or p.Gly44Val in the ALG1 gene. Transferrin isoform analysis showed a CDG type I pattern, consistent with a glycosylation disorder. This case emphasizes the importance of early clinical suspicion, metabolic screening, and genetic testing in infants with unexplained recurrent seizures, syndromic features, and multisystemic involvement.

## Introduction

Congenital disorder of glycosylation type Ik (CDG Ik) is a rare, severe, and typically rapidly progressive inherited metabolic disorder that falls under the category of N-linked glycosylation defects. It was independently discovered by three groups in the year 2004 [1-3]. This autosomal recessive genetic condition is caused by pathogenic variants in the *ALG1* gene, which encodes the critical enzyme beta-1,4-mannosyltransferase. This enzyme is essential for one of the first steps in N-glycan assembly, catalyzing the addition of the first mannose sugar unit to the nascent oligosaccharide chain anchored to dolichol phosphate in the endoplasmic reticulum [4]. When the *ALG1* activity is compromised, the incomplete sugar structure impairs the function and stability of numerous vital glycoproteins across multiple organ systems, resulting in the disease's pronounced multisystemic presentation [5-9]. We report a ten-month-old female with CDG type Ik, presenting with recurrent seizures, global developmental delay, hypotonia, and dysmorphic features. Diagnosis was possible on whole-exome sequencing (WES) and transferrin isoform analysis. This is the second published case report of CDG Ik from India to the best of our knowledge, and it will help increase awareness about this ultra-rare condition. Also, identification of the same pathogenic variant in the *ALG1* gene as the previously published case indicates a founder effect in India.

## Case presentation

**Figure 1:**
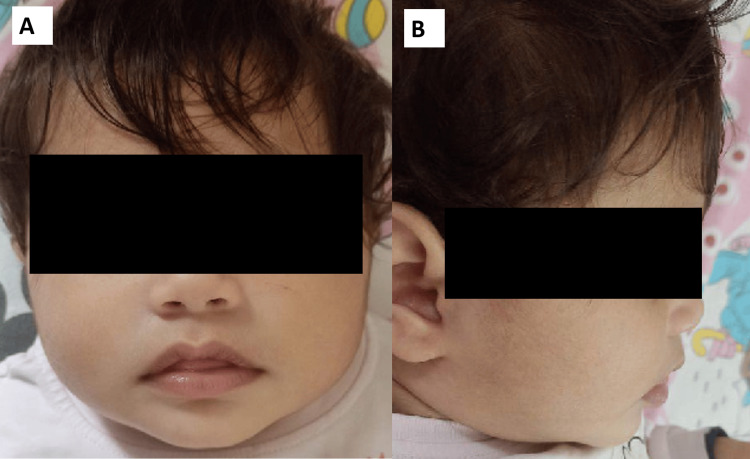
Facial dysmorphism seen in the patient. (A) Facial features of the patient (frontal view) demonstrating microcephaly, round face, mild coarsening of features, epicanthic folds, depressed nasal bridge, and a prominent tongue, and (B) lateral view of the patient’s face showing large, low-set ears with flat midface. Informed consent for publishing facial photos has been taken from the patient's family.

We describe a ten-month-old female infant, the first child of a third-degree consanguineous marriage, who presented with recurrent generalized tonic-clonic seizures (GTC) lasting a few minutes with no residual focal neurological deficits, initially fever-triggered. The first episode was at six months of age, followed by three episodes of afebrile GTC once every four weeks, which progressed to daily five to six episodes of multifocal seizures. The child was born via normal vaginal delivery, weighing 2.7 kg, without any significant perinatal illness. Developmentally, the child was significantly delayed with a developmental age of approximately three months. At ten months of age, she had only partial neck control, was unable to sit unsupported, and had not achieved babbling. Her social interaction was limited to recognizing her mother. On examination, her anthropometry was as follows: weight 8.3 Kg (43rd centile), total length 72 cm (57th centile), and head circumference 40.7 cm (0.3rd centile) (microcephaly). The child had dysmorphic features-a round face, coarsening of facies, epicanthic folds, depressed nasal bridge, flat midface, a prominent and protruded tongue, low-set ears, slender fingers, and floppy limb posture (see Figures 1A, 1B).

Comorbidities associated with CDG Ik, including peripheral neuropathy, coagulation or bleeding tendencies, chronic diarrhea, constipation, edema due to protein-losing enteropathy, hepatomegaly, liver dysfunction, renal dysfunction, immunodeficiency, feeding difficulties or vomiting, were absent. Neurological examination revealed generalized hypotonia with brisk deep tendon reflexes, but no spasticity or cranial nerve deficits.

**Figure 2:**
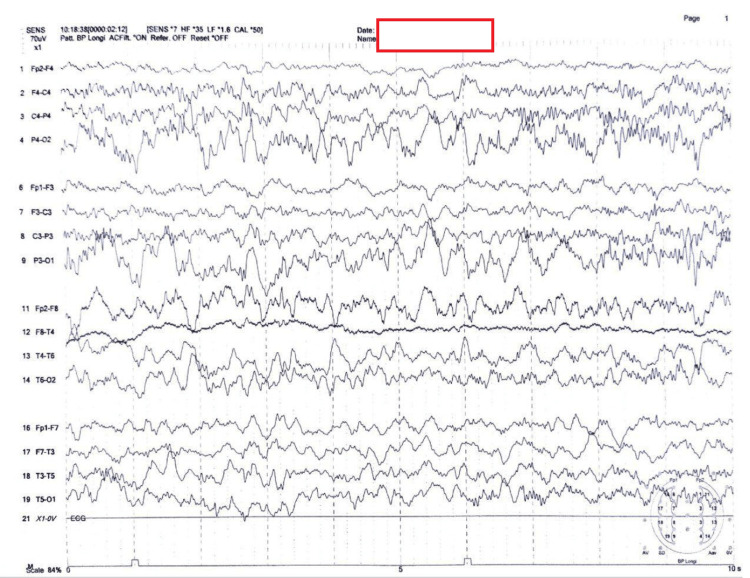
Electroencephalograph of the patient. Electroencephalograph of the patient demonstrating generalized seizure or epileptiform activity.

Magnetic resonance imaging (MRI) of the brain was normal. Brainstem evoked response audiometry (BERA) and fundus examination were unremarkable, indicating no evidence of hearing or visual impairment. Electroencephalography (EEG) revealed generalized epileptiform activity. The EEG tracing showed a lack of rhythmic or organized background activity. The tracing is dominated by high-voltage, multifocal spikes and sharp waves that appear randomly across nearly all leads. The amplitudes are notably high, and the waveforms frequently exceeded the grid lines. The discharges are non-synchronous and vary significantly in morphology and location. The abnormalities are generalized but particularly prominent in the posterior and central leads (P4-O2, P3-O1) (see Figure 2). 

Two-dimensional echocardiography showed a left main coronary artery (LMCA) to cameral fistula, a small 1-2 mm muscular ventricular septal defect (VSD), and a mild aortic gradient. The electrocardiogram (ECG) showed right-axis deviation. Thyroid evaluation, namely serum thyroid-stimulating hormone (TSH), T3, and T4 levels, was within normal limits (see Table 1).

**Table 1:** Thyroid function tests in the child. Thyroid function tests show normal values.

Test parameter	Patient value	Normal range (2.5th–97.5th centile)	Standard units
Thyroid-stimulating hormone (TSH)	2.5	1.12-8.21	mIU/L
Free thyroxine (FT4)	2.4	0.9-2.8	nanogram/dL
Free triiodothyronine (FT3)	3.8	1.5-6.0	picogram/ml
Total T4	6.2	4.3-7.6	microgram/dL

The abdominal ultrasound was unremarkable. However, transferrin isoform analysis (HPLC) showed a type I congenital disorder of glycosylation disease pattern consistent with elevated asialotransferrin, disialotransferrin, and decreased tetrasialotransferrin and normal trisialotransferrin and pentasialotransferrin, a profile indicative of an N-glycosylation defect (see Table 2).

**Table 2:** Results of serum transferrin sialic acid isoform analysis in the serum of the index child. The results suggest elevated asialotransferrin and disialotransferrin and low tetrasialotransferrin (mature isoform), which are consistent with the pattern observed in patients with congenital disorder of glycosylation type I.

Test (sialic acid isoform in serum)	Result (normal relative percentage)	Reference (in %)
Asialotransferrin	12.68	0-1.5
Disialotransferrin	43.14	0.4-2.4
Trisialotransferrin	1.77	1.6-6.6
Tetrasialotransferrin	33.78	81-94
Pentasialotransferrin	8.62	3-15

Liver tests were normal (see Table 3).

**Table 3:** Liver function tests in the index child. The results show normal values.

Serum test component	Result	Reference range (1-12 months)	Units
Alanine aminotransferase (ALT)	24	12-45	U/L
Aspartate aminotransferase (AST)	38	9-80	U/L
Alkaline phosphatase (ALP)	285	150-420	U/L
Gamma-glutamyl transferase (GGT)	18	5-32	U/L
Total bilirubin	0.6	<2.0	mg/dL
Albumin	4.2	3.5-5.0	g/dL
Total protein	6.4	5.5-7.0	g/dL

 Renal tests were normal (see Table 4). 

**Table 4:** Renal function tests in the index child. The results of renal function tests in the child are normal.

Serum test component	Result	Reference range (0-11 months)	Units
Creatinine	0.31	0.17-0.42	mg/dL
BUN (urea nitrogen)	12	5-18	mg/dL
Sodium	139	130-147	mmol/L
Potassium	4.8	3.4-5.6	mmol/L
Chloride	104	95-108	mmol/L
Calcium (Total)	9.6	8.7 – 11.0	mg/dL

Urine examination was normal (see Table 5).

**Table 5:** Urine examination in the child. The results of the urine examination in the child were normal.

Urine parameter	Result	Normal finding
Color/clarity	Straw/clear	Straw/clear
Specific gravity	1.008	1.002-1.015
pH	6	5.0-7.0
Leukocyte esterase	Negative	Negative
Nitrite	Negative	Negative
Protein	Negative	Negative
Glucose/ketones	Negative	Negative
Microscopic white blood cells (WBCs)	1-2	<20 cells/µL
Microscopic red blood cells (RBCs)	0-1	<5 cells/µL

Lymphocyte CD4 and CD8 subsets were normal (see Table 6).

**Table 6:** Blood lymphocyte subset analysis (CD4 and CD8) in the index child. Results of lymphocyte subset analysis (CD4 and CD8) in the child were normal.

Blood test component	Result	Reference range (6-12 months)	Units
Absolute CD4+ count	2,450	1,400-4,300	cells/µL
CD4%	42%	31%-56%	%
Absolute CD8+ count	1,100	400-2,100	cells/µL
CD8%	21%	12%-28%	%
CD4/CD8 ratio	2.2	1.0-3.5	Ratio

The child's serum immunoglobulin profiles were normal (see Table 7).

**Table 7:** Serum immunoglobulin profile in the child. The serum immunoglobulin profile in the child was normal.

Serum test component	Result	Reference range (7-12 months)	Units
IgG	580	300-900	mg/dL
IgA	35	15-85	mg/dL
IgM	72	40-150	mg/dL

**Figure 3:**
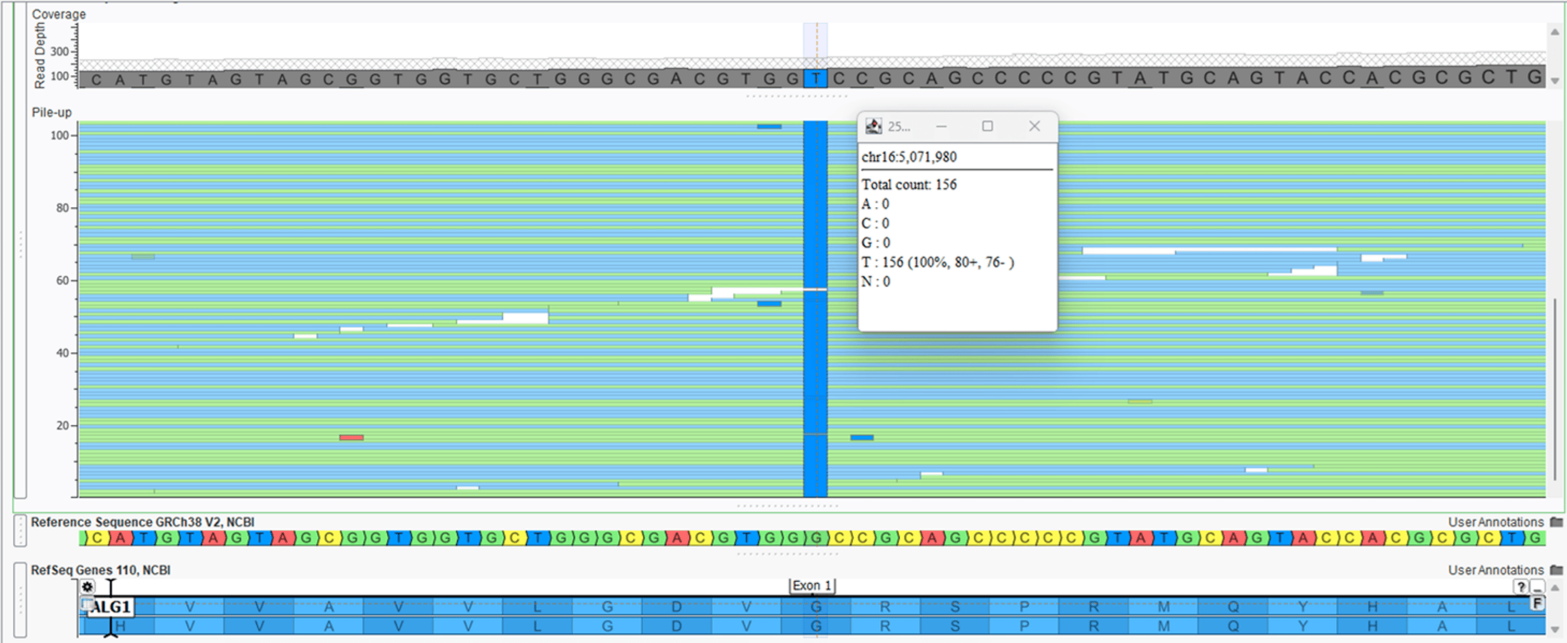
Homozygous variant in the ALG1 gene in the index case. Screen-capture of the Integrated Genomic Viewer (VarSeq Software; Golden Helix Incorporation, USA) of the binary alignment map file of the exome data in the index child showing a homozygous variant in the *ALG1* gene (chr16-5071980 G>T or c.131G>T or p.Gly44Val, transcript ID NM_019109.5, dbSNP ID:rs1336732262) located on chromosome 16p13.3.

Whole-exome sequencing (WES) (20321 nuclear genes and 37 mitochondrial genes) (Twist 2.0 capture assay with a mitochondrial probe set added) (from Twist Biosciences Inc., South San Francisco, California, USA) was performed for the index patient at an accredited laboratory (SN Gene Lab Pvt Ltd). This assay detects both single-nucleotide variants (SNVs) and copy number variants (CNVs). WES identified a homozygous missense variant in exon 1 of the *ALG1* gene (chr16-5071980 G>T or c.131G>T or p.Gly44Val, transcript ID NM_019109.5, dbSNP ID:rs1336732262) located on chromosome 16p13.3 (see Figure 3). 

The variant is likely pathogenic by the American College of Medical Genetics and Genomics criteria (criteria satisfied: PS1, PM2, PP3, PP4) [10].

**Figure 4:**
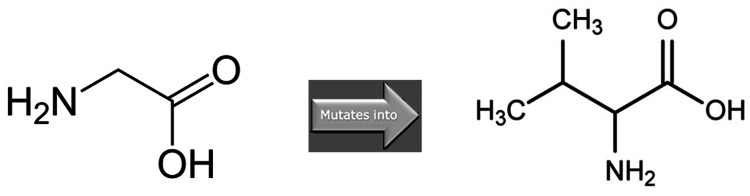
Glycine to valine missense variant. The figure shows line diagrams representing the backbone of amino acids. Glycine (on left) has been substituted by valine (on right). The mutant residue is bigger and more hydrophobic than the wild-type residue. Figure was created by the Have Your Protein Explained (HOPE) insilico tool (Center for Molecular and Biomolecular Informatics (CMBI), Radboud University Medical Center (Radboudumc), Nijmegen, The Netherlands).

The Have Your Protein Explained (HOPE) insilico tool (Center for Molecular and Biomolecular Informatics (CMBI), Radboud University Medical Center (Radboudumc), Nijmegen, The Netherlands) was used to analyze the structural effects of the p.Gly44Val variant in the ALG1 gene [11]. No structural information is known for this protein. No solved 3D structure or modeling template could be found. Hence, HOPE used annotations from the UniProt database (entry Q9BT22) and predictions from the Reprof software (Rost Lab, Technical University of Munich (TUM), Germany) for mutation analysis. The analysis reported the following. The mutant residue is bigger and more hydrophobic than the wild-type residue (see Figure 4). 

**Figure 5:**
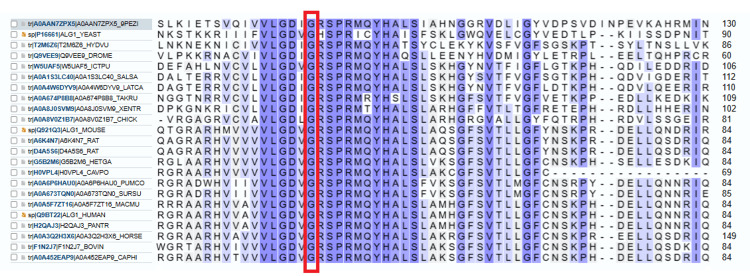
The glycine 44 residue of the ALG1 protein is conserved across different species. Multiple sequence alignment of ALG1 protein in different species shows that glycine (G) is conserved at this position (highlighted in red) across different species such as, from top to bottom, are *Elasticomyces elasticus*, *Saccharomyces cerevisiae*, *Hydra vulgaris*, *Drosophila melanogaster*, *Ictalurus punctatus* (Channel catfish), *Salmo salar* (Atlantic salmon), *Lates calcarifer* (Barramundi), *Takifugu rubripes* (Japanese pufferfish), *Xenopus tropicalis* (Western clawed frog), *Gallus gallus* (Chicken), *Mus musculus* (Mouse), *Rattus norvegicus* A6K4N7 (Rat), *Rattus norvegicus* D4A5S6 (Rat), *Heterocephalus glaber* (Naked mole rat), *Cavia porcellus* (Guinea pig), *Puma concolor* (Mountain lion), *Suricata suricatta* (Meerkat), *Macaca mulatta* (Rhesus macaque), *Homo sapiens* (Human), *Pan troglodytes* (Chimpanzee), *Equus caballus* (Horse), *Bos taurus* (Bovine), *Capra hircus* (Goat).

The mutation is located within the lumenal repeat region of the protein. The missense mutation possibly perturbs this region and its function. Substitution of glycine, the most flexible of all amino acids, with another may lead to secondary changes in protein structure. This variant's MetaRNN score (pathogenicity prediction score) is 0.9556339. Conservation studies showed that glycine occupies this position in all species (100% conservation) (see Figure 5).

In summary, we describe a child with facial dysmorphism, global developmental delay, and drug-resistant epilepsy diagnosed to have CDG Ik based on DNA testing and serum transferrin sialic acid isoform analysis. The overall prognosis remains guarded, with treatment aimed at improving quality of life. This child was started on oral levetiracetam at a dose of 25 mg/kg/day, which was increased to 60 mg/kg/day. Despite this, she developed drug-resistant epilepsy and required the addition of phenobarbitone (7 mg/kg/day) and topiramate (1 mg/kg/day). On follow-up presently, she had responded to the anti-epileptic medications except for two episodes of generalized seizures, which responded to nasal midazolam (0.5 mg per puff, dose 0.2 mg/kg/dose) administered by the parents. Supportive therapies, including physiotherapy, developmental stimulation, and nutritional management, were initiated in view of global developmental delay and hypotonia. The family was provided with genetic counseling, and options for prenatal diagnosis and carrier testing for future pregnancies were discussed. Recommended follow-up includes annual monitoring of growth, neurodevelopment, liver and renal function, coagulation profile, and thyroid status. Early initiation of physical, occupational, and speech therapy is critical to optimize developmental outcomes [5-9].

Written informed consent was obtained from the patient’s parents for the reporting of this case. The study was conducted in accordance with the principles of the Helsinki Declaration. The study was approved by the Ethics Committee of Dr. D. Y. Patil Medical College, Hospital and Research Center, Pimpri, Pune (DYPV/EC/612/2020).

## Discussion

Congenital disorders of glycosylation (CDG) are a group of rare, inherited metabolic disorders caused by defects in the glycosylation of proteins and lipids, with variants identified in over 100 unique genes. The *ALG1* gene is located on chromosome 16p13.3 and encodes β1,4-mannosyltransferase, a key enzyme required for the assembly of dolichol-linked oligosaccharide precursors during early N-glycan biosynthesis [1-3]. To date, the ClinVar database lists 117 pathogenic and 65 likely pathogenic variants in the *ALG1* gene, with the missense mutation c.773C>T (p.S258L) being one of the most frequently described [1,2,12]. The disorder follows an autosomal recessive inheritance pattern, with a 25% recurrence risk in each pregnancy for carrier parents [5-9].

The clinical presentation of ALG1-CDG is highly variable, ranging from severe neonatal-onset forms with early lethality to milder phenotypes with survival into adolescence or adulthood [5-8]. Neurological involvement is the predominant feature, with hypotonia, developmental delay, progressive microcephaly, seizures, and movement disorders being the most common manifestations [8]. Systemic features include failure to thrive along with coagulation defects, immunodeficiency with recurrent infections, hepatic involvement such as hepatomegaly and portal hypertension, renal disease including nephrotic syndrome, and cardiac problems like congenital heart defects, such as ventricular septal defect (VSD), coronary artery anomalies, and cardiomyopathy in rare cases [5-9]. Skeletal findings such as contractures, arachnodactyly, scoliosis, and fleshy finger pads have also been reported. Ophthalmic abnormalities, including strabismus, nystagmus, and, in some patients, progressive vision loss due to optic atrophy, have been described. Deafness has also been reported [5-9]. In our patient, we found no evidence of hearing or visual impairment.

Prenatal features such as hydrops fetalis, arthrogryposis, hepatosplenomegaly, polyhydramnios, and reduced fetal movements have been observed in some cases [8]. Severe neonatal presentations may be associated with early mortality within the first weeks or months of life [8]

The largest case series, published by Ng et al., described 39 previously unreported patients from 32 families and identified 26 novel pathogenic variants [8]. Their study also demonstrated the diagnostic utility of a unique xeno-tetrasaccharide biomarker (NeuAc-Gal-GlcNAc₂). Bosnyak et al. reported a case where severe *ALG1* deficiency presented with normal transferrin glycosylation, highlighting potential diagnostic pitfalls in relying solely on biochemical markers [13]. Öncül et al. in 2017, described a 15-month-old Turkish girl with ALG1-CDG who exhibited hypotonia, developmental delay, seizures, dysmorphic features, failure to thrive, and coagulation abnormalities. Transferrin isoelectric focusing revealed a CDG type I pattern, reinforcing the need for high clinical suspicion in multisystem and atypical presentations [14]. Similarly, recent literature continues to expand the phenotypic and genotypic spectrum of ALG1-CDG [15,16]. More recently, Khaghani et al. (2025) reported the first cases of ALG1-CDG from Iran. They presented three individuals with early-onset seizures, developmental delay, and cognitive impairments, confirmed via whole-exome sequencing (WES) [17].

The variant reported in our patient has been previously reported in the ClinVar database (year 2022) in a homozygous state in an Indian patient with the following clinical features: oligohydramnios, global developmental delay, facial dysmorphism, optic atrophy, cerebral visual impairment, myoclonic seizures, hypotonia, delayed myelination, type I CDG pattern on transferrin isoform testing [18].

Diagnosis of ALG1-CDG typically involves biochemical screening through transferrin isoform analysis, revealing elevated disialotransferrin and reduced tetrasialotransferrin, consistent with a type I glycosylation defect. However, as some patients may present with normal profiles, molecular confirmation via next-generation sequencing remains the gold standard [13].

## Conclusions

We report a confirmed case of CDG type Ik, diagnosed via exome sequencing and transferrin isoform analysis. This suggests the importance of timely exome sequencing in patients with global developmental delay, dysmorphism, or resistant epilepsy. Early diagnosis of this disorder by timely exome sequencing enables precise diagnosis, appropriate management, which is primarily supportive, including seizure control with anti-epileptic medications, physiotherapy, nutritional support, regular monitoring for multisystem involvement, genetic counseling, and prenatal and pre-implantation diagnostic options for future pregnancies. Extended family screening for carriers is also important to prevent recurrences of this disease in future progeny. Identification of the p.Gly44Val variant in the *ALG1* gene in the second unrelated case in India may point towards a founder effect.
